# Ancient DNA suggests anaemia and low bone mineral density as the cause for porotic hyperostosis in ancient individuals

**DOI:** 10.1038/s41598-023-33405-7

**Published:** 2023-04-28

**Authors:** Manuel Ferrando-Bernal

**Affiliations:** Unaffiliated, Barcelona, Spain

**Keywords:** Genetics, Medical genetics

## Abstract

Porotic hyperostosis (PH) is a disease that had high prevalence during the Neolithic. Several hypotheses have been suggested to explain the origin of the disease, such as an iron deficiency diet, low B12 intake, malaria caused by *Plasmodium* spp., low haemoglobin levels or low vitamin D levels. None of these hypotheses have been tested genetically. Here, I calculated different genetic scores to test each hypothesis. Additionally, I calculated a genetic score of bone mineral density as it is a phenotype that seems to be selected in ancient Europeans. I apply these genetic scores on 80 ancient samples, 33 with diagnosed PH. The results seem to suggest anaemia and low bone mineral density as the main cause for this disease. Additionally, Neolithic individuals show the lowest genetic risk score for bone mineral density of all other periods tested here, which may explain the highest prevalence of the porotic hyperostosis during this age.

## Introduction

**Figure 1:**
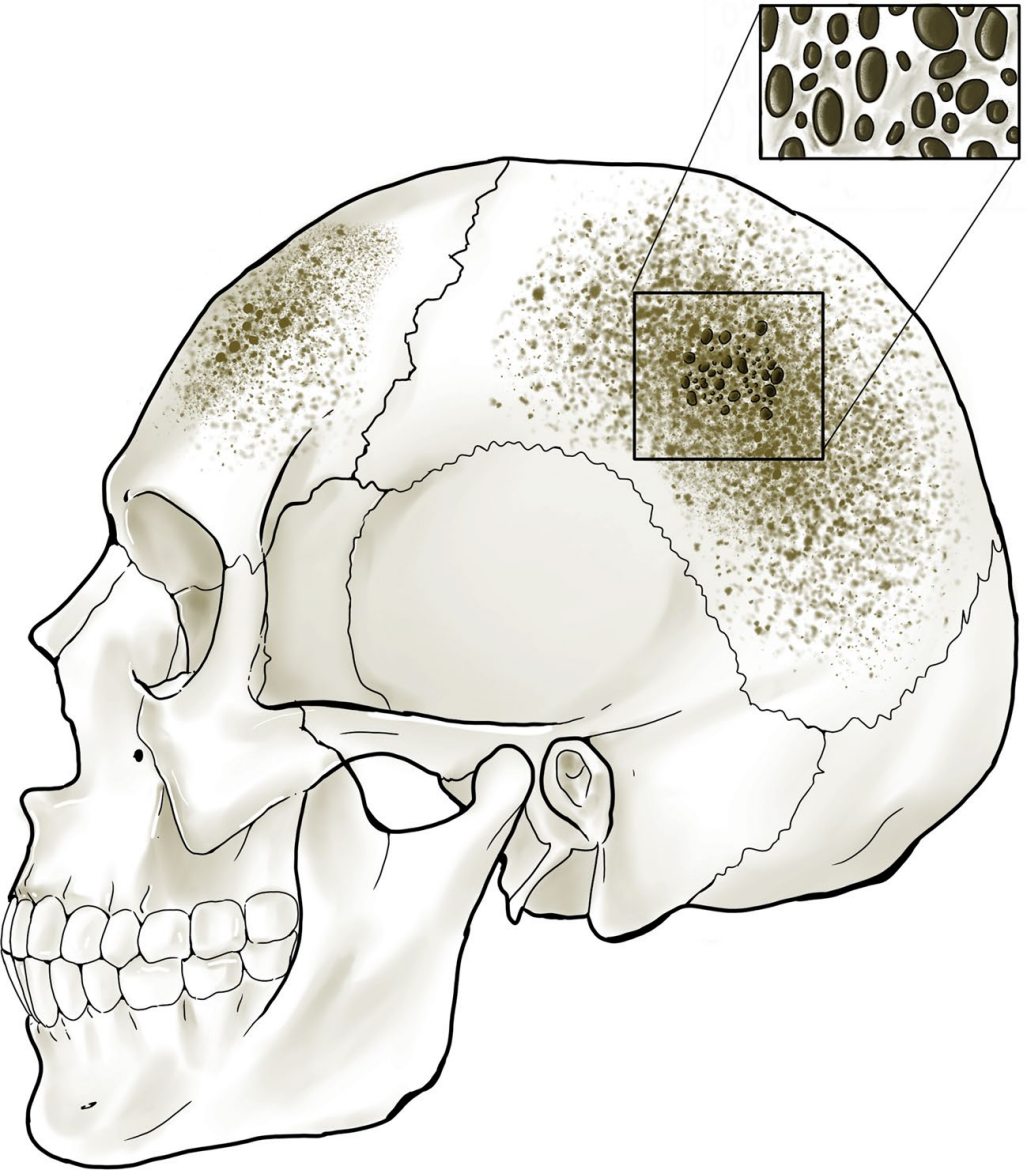
Porotic hyperostosis is characterised by thicker skull bones with an increased porous area caused by an expansion of the cranial diploë.

Paleopathology is the study of disease in ancient organisms. Unsurprisingly, the majority of studies focus on past pathologies affecting ancient humans^1,2^. Due to the nature of ancient remains, bone disorders, such as bone cancer, rickets or bone traumatism, are the most studied in paleopathology. One of the best claimed diseases affecting past populations is Porotic hyperostosis (PH), a skeletal disorder characterised by bony lesions on the bones of the skull due to an expansion of the cranial diploë^3^ (Fig. 1). This condition has been observed in ancient individuals belonging to several populations^4–8^, but mainly in individuals dating to the Neolithic period^2,9–11^, reaching 43% of the individuals in some populations^11^. The lesions are typically found on the frontal and parietal bones, and can range from small pits to large lesions. The main cause behind this disease is not well known, likely due to its low prevalence in modern times^12,13^. There has been a lot of speculation and many hypotheses have been proposed, such as, low vitamin D levels that may lead to thinner skulls due to its implication in skeletal mineralization^14^, or even nutritional deficiency at weaning during childhood^15^, as deficiency of vitamin B12 may cause red blood cells abnormalities, leading to a need for a diploë expansion to increase in the red cell production^15^. However these hypotheses have not been scientifically tested. However, since the 1950s, anaemia has been thought as the main cause for PH^15^. For example, it has been linked to infectious disease, such as malaria, as the parasite *Plasmodium falciparum* might have caused anaemia by an excess of red blood cell destruction^16^. Other researchers suggest that anaemia caused by thalassemia may be the main cause of PH^13,16^. Even an iron-deficiency anaemia, caused by a lack of dietary iron, poor absorption of iron, or increased demand for iron due to high levels of physical activity^14^ has been proposed. Nevertheless, this last hypothesis was rebutted as iron deficiency may not nourish the large red blood cell production that causes the narrow expansion^17^. Additionally, Eurasians seem to have been selected for low bone mineral density (BMD) levels during the last thousand years^18,19^, which may lead to particularly thin bones in some individuals.

To date, studies of PH in ancient individuals used indirect evidence to test for a particular hypothesis or another. However, present-day technologies and more than three decades of study of ancient DNA allow us to test them genetically. To date, we have been able to recover the genomes of thousands of ancient samples and test many hypotheses that have been unanswered for decades, such as our past relationships with other *Homo* species^20,21^, connections among ancient and modern individuals^22^ and the selective pressures of particular human traits^23^. While numerous studies focused on the implication of breeding with Neanderthals and Denisovans on the development of different diseases in modern individuals^24^, the contribution of ancient DNA to paleopathology has mainly focused on infectious diseases, such as confirming *Yersinia pestis* as the pathogenic agent of ancient plagues^25^, comparing the genes related to pathogenic resistance in ancient populations^26^, determining the oral and microbiome of ancient individuals^27^, and studying the prevalence of malaria in ancient times^28^. Yet, ancient DNA still has a huge potential to elucidate the genetic architecture of diseases affecting ancient populations^29^. With this idea in mind, I calculated different genetic scores using hundreds of SNPs related to specific phenotypes and applied it to 80 ancient genomes for which we can differentiate if they were affected or not by PH. These genetic scores were then used to test for the different hypothesis proposed for the origin of PH (see Table 1 for the hypothesis tested and its predictions).

**Table 1:** Hypothesis and predictions for the origin of PH.

Proposed hypothesis for porotic hyperostosis	Predictions
1. Caused by low bone mineral density levels	Individuals with PH may have lower bone mineral density than unaffected individuals
2. Caused by low vitamin D levels^14^	PH individuals may have higher skin pigmentation than unaffected individuals
3. Anaemia caused by low iron intake^15^	Changes in SNPs related to Iron metabolism among individuals with and without PH
4. Megaloblastic anaemia caused by low vitamin B12 intake^15^	Changes is SNPs related to vitamin B12 metabolism among individuals with and without PH
5. Anaemia caused by malaria^16^	Changes in SNPs related to malaria resistance among individuals with and without PH
6. Anaemia caused by low haemoglobin levels^13,16^	PH individuals may have lower haemoglobin concentration than unaffected individuals

The results suggest anaemia as the main cause for PH. Specifically, an anaemia that was caused by a genetic tendency for low haemoglobin levels together with genetic architecture for low bone mineral density. Specifically, Neolithic individuals show low levels for BMD compared to previous and recent populations which may explain why PH had the highest prevalence levels during this period.

## Material and methods

### Databases

#### SNPs related to BMD

Bone mineral density (BMD) is the amount of minerals (such as calcium) deposited in the bones. Many factors influence the levels of BMD. It can be influenced by environmental cues like the levels of diary activity or the diet, but also by genetic factors^18,19^. Eurasians tend to have lower BMD compared to African populations. This has been interpreted as the result of selective events since the Out-of-Africa^18,19^. In order to see if individuals with PH had lower BMD, I used the most broad study of SNPs related to BMD available currently, with more than 1000 SNPs^30^. The datasets used here recover 233 of these SNPs (see Supplementary Table 1). These SNPs were used to create a genetic score of the BMD in the ancient individuals.

#### SNPs related to skin pigmentation

Europeans evolved low levels of melanin in the skin cells. Ancient DNA studies suggest that this selection increased after the Agricultural Revolution^31,32^. While the main evolutionary cause for this phenomenon remains obscure, low melanin production has been related to increased levels of vitamin D in blood. Vitamin D is synthesised by the influence of UV-radiation from the sun, and skin tones with higher melanin block more of this UV-radiation^32^. Vitamin D is needed to absorb calcium in the gut and is fundamental for the development of healthy immune and skeletal systems, including normal levels of mineral deposition in the bones^32^.

As selective pressure for light skin increased during the Neolithic^31,32^, early farmers may have had lower vitamin D levels than recent populations (such as Bronze, Copper or Iron Age individuals) leading to a lower capacity to synthesise vitamin D. This may explain why the prevalence of PH was higher during the Neolithic than during recent times. To assess this, I used a broad dataset of SNPs related to skin pigmentation (that has previously applied to ancient DNA studies^32^) to generate a genetic score of skin pigmentation in the ancient samples. 173 of 175 SNPs related to skin pigmentation were recovered in the dataset used here (see Supplementary Table 2).

#### SNPs related to iron metabolism

Iron is needed to produce haemoglobin, a protein that transports oxygen molecules throughout our body. Thus, iron deficiency may lead to anaemia and it has been proposed that it may be the cause of the expansion of the cranial diploë. However, this hypothesis does not seem to be plausible as low iron levels cannot support the increased need for red blood cell production that follows the expansion of the bone marrow^17^. Several PH studies suggested that a diet low in iron may have followed the transition from hunter-gathering to farming. Additionally, pathogens can retain the iron from blood, reducing its levels, and it has been suggested that an increase of pathogenic diseases during the early Neolithic caused a reduction in the levels of iron in early neolithic individuals. This may explain why PH had a higher incidence than in recent times. All these scenarios should have triggered changes in the genes related to iron metabolism. To test for this notion I studied changes in the genetic score values in 8 SNPs^33^ related to iron metabolism in Europeans that can be recovered in the ancient dataset used here (see Supplementary Table 3).

#### SNPs related to vitamin B12 metabolism

Vitamin B12 is needed for a normal formation of red cells. Vitamin B12 deficiency during weaning was speculated as a possible cause for PH^15^. Additionally, an animal diet is richer in Vitamin B12 compared with a diet rich in vegetables, so a ransition from hunter-gathering to farming may then have led to a diet low in vitamin B12 and an impaired formation of red blood cells. At the beginning of the Neolithic the variety of vegetable food decreased together with a reduction of meat consumption^34,35^ possibly leading to malnutrition and diseases such as anaemia or scurvy^36^. Thus, this behavioural change may explain why PH had a higher prevalence in the Neolithic^2,9–11^ than in previous times (Mesolithic and Palaeolithic). It also can explain why PH was more common in the Neolithic than in more recent times (Bronze, Iron and Copper Ages) as the number of domesticated animal species increased and were included in the diet. This could have triggered changes in vitamin B12 metabolism. Here, I used a genetic score with four SNPs^37^ related to vitamin B12 metabolism that can be recovered in the database of ancient samples used here (see Supplementary Table 4).

#### SNPs related to malaria resistance

The prevalence of malaria in ancient populations is still today a controversial subject^28,38^. The *Plasmodium falciparum* parasite destroys red cells during its life cycle, causing a disease known as malaria, characterised by anaemia. Several studies point toward malaria as the cause for PH. To test this hypothesis, I used 104 SNPs related to malaria resistance^39^, together with 22 SNPs from^28^. As this phenotype can be caused by a single genomic variant, I decide not to conduct a genetic risk score in this phenotype but a FST analysis as in Gelabert et al.^28^, where the authors used 22 SNPs to look for evidence of malaria as a selective agent in ancient populations. 77 of these SNPs could be recovered in the ancient datasets (see Supplementary Table 5).

#### SNPs related to haemoglobin levels

Haemoglobin is the protein that binds oxygen molecules inside red blood cells. Low levels of haemoglobin lead to anaemia, possibly causing PH. Low levels of haemoglobin have been associated with genetic variants^40^ such as in thalassemia, a genetic condition affecting one of the genes for the α of β globin^40^. Interestingly, thalassemia is common around the Mediterranean Sea populations and has been referred to as Mediterranean anaemia. To test if low haemoglobin levels are the main cause for PH, I conducted a genetic score with SNPs related to haemoglobin levels in inhabitants of Sardinia (in the western part of the Mediterranean Sea)^41^. Five SNPs could be recovered in the ancient dataset, one of them affecting the gene for the β globin (see Supplementary Table 6).

### Genetic risk score estimation

Many phenotypes are influenced by multiple genetic variants, as in the case of bone mineral density and skin pigmentation. All the additive effects of each variant can be aggregated together into a simple measure, known as genetic risk score^44^. Genetic risk score can be understood as a value for the genetic risk of an individual to develop a particular phenotype. It has been continuously applied to ancient individuals, for example the two datasets used here independently used a genetic risk score to calculate the height of the individuals based on height related SNPs^42,43^. Other studies applied genetic risk scores to measure skin pigmentation as in Du, J. & Mathieson, I. 2020^32^, or the likelihood to develop attention/deficit hyperactivity disorder in Neanderthals and ancient Europeans^45^, as well as hundreds of different phenotypes^46^. The genetic risk score here was calculated as the percentage in each individual of SNPs related to: (1) high bone mineral density, (2) darker skin pigmentation, (3) high iron levels, (4) vitamin B12 levels and (4) high haemoglobin levels (see Supplementary Tables 10 and 11).

### FST analysis for malaria resistance

FST analysis for the 77 malaria resistance SNPs were calculated with PLINK^47^ (see Supplementary Table 5).

### Principal Component Analysis

A Principal Component Analysis (PCA) has been generated with PLINK^47^ to test if healthy individuals cluster differently compared with affected individuals. For the analysis all markers that were missing in at least 10% of the individuals (8 individuals) and all markers with a MAF higher than 0.05 were removed. Finally, 772,964 SNPs were left for the PCA analyses.

### Statistical analysis

All statistical analyses were conducted in R Studio^48^. Wilcoxon Mann Whitney test was used to analyse differences in individuals with and without porotic hyperostosis among the different genetic scores. Pearson correlations were used to study differences among the genetic scores of BMD levels through time.

### Figures and plots

Figure 1 was generated in Sketchbook^49^ and the credits correspond to Paula Roig Ferrando. Other figures were conducted in RStudio^48^.

## Results and discussion

### Porotic hyperostosis is common in ancient individuals with a genetic risk for low bone mineral density and low haemoglobin levels

**Figure 2:**
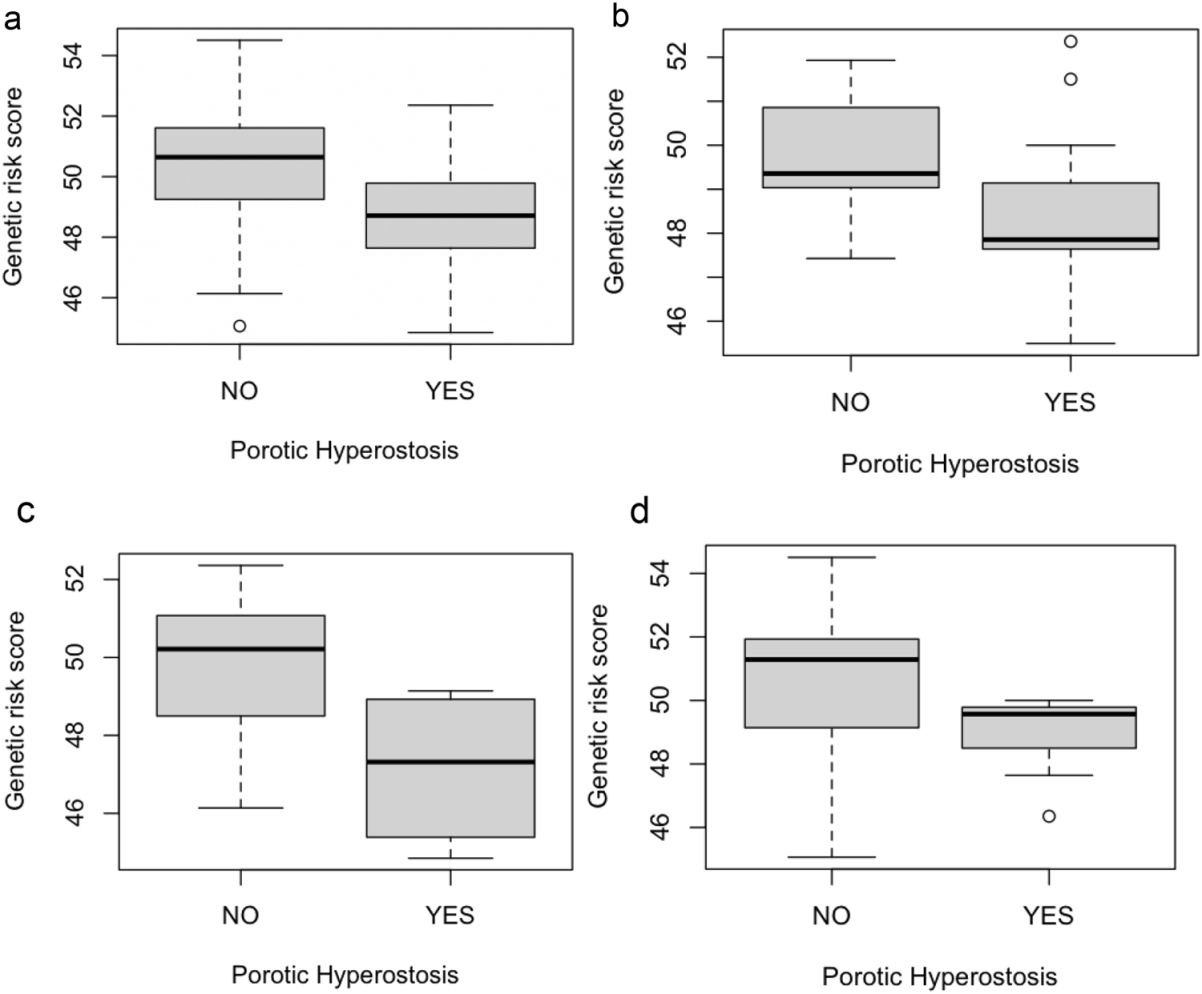
Individuals with porotic hyperostosis tend to have lower frequency of high BMD related alleles in all observed ages. (**a**) Whole dataset, p value = 0.00007605. (**b**) Neolithic p value = 0.1041. (**c**) Bronze Age, p value = 0.05366. (**d**) Copper Age, p value = 0.03521.

**Figure 3:**
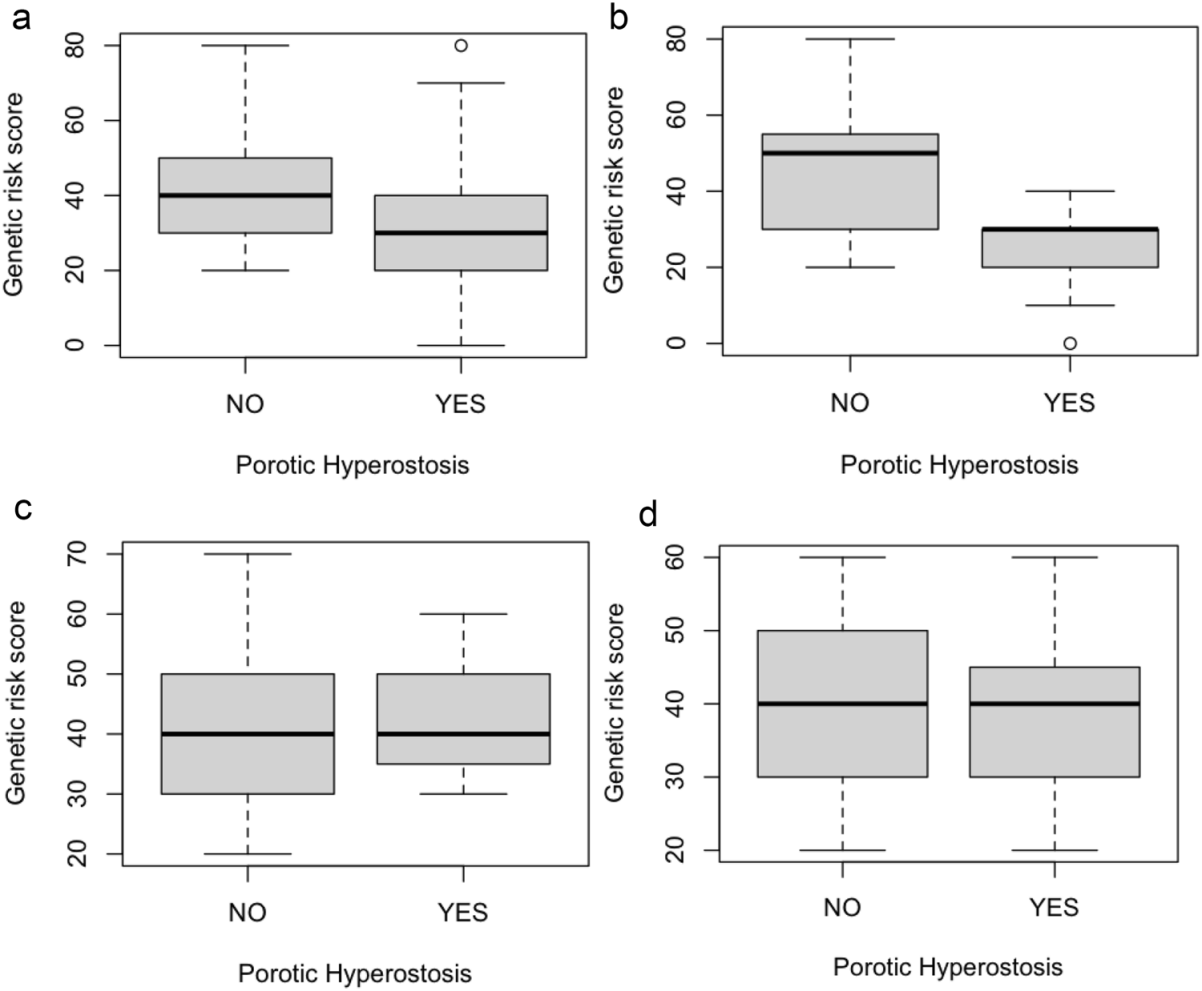
Individuals with porotic hyperostosis tend to have lower genetic risk scores for haemoglobin levels, specifically during the Neolithic. (**a**) Whole dataset, p value = 0.02489. (**b**) Neolithic p value = 0.03555. (**c**) Bronze Age, p value = 1. (**d**) Copper Age, p value = 0.8111.

When comparing the individuals with and without PH, I detected significant statistical differences for the genetic risk scores of BMD (see Fig. 2) and Haemoglobin levels (see Fig. 3) (the p values for Mann Whitney Wilcoxon tests were 0.00007605 for the BMD and 0.02489 for the Haemoglobin levels), while the genetic risk scores of skin pigmentation, iron levels, vitamin B12 levels did not show statistical differences between the two groups. Individuals with PH had lower genetic risk scores for a high BMD during all ages from the Neolithic to the Copper Age (see Fig. 2). However, the genetic risk scores of Haemoglobin levels show statistical differences only when all individuals are compared and during the Neolithic, and lose significance during the Bronze and the Copper Ages (Fig. 3), this may be due to the lack of data as only 5 related SNPs could be used in this analyses. Palaeolithic individuals were not used in this analysis due to the low number of samples. Iron Age samples were not used as no individual from this period was found to have PH.

These results do not support the hypothesis that PH was caused by low vitamin D levels in darker individuals or changes in the iron or in the vitamin B12 metabolism. Instead, the results support the hypothesis that anaemia caused by low haemoglobin levels could be responsible for PH, together with a genetic architecture for low bone mineral density. Interestingly, low BMD correlates with low haemoglobin levels^50–52^. A possible explanation for this is that anaemia caused by low haemoglobin levels may correlate with a genetically porous bone to allow for an expansion of the diplöe.

**Figure 4:**
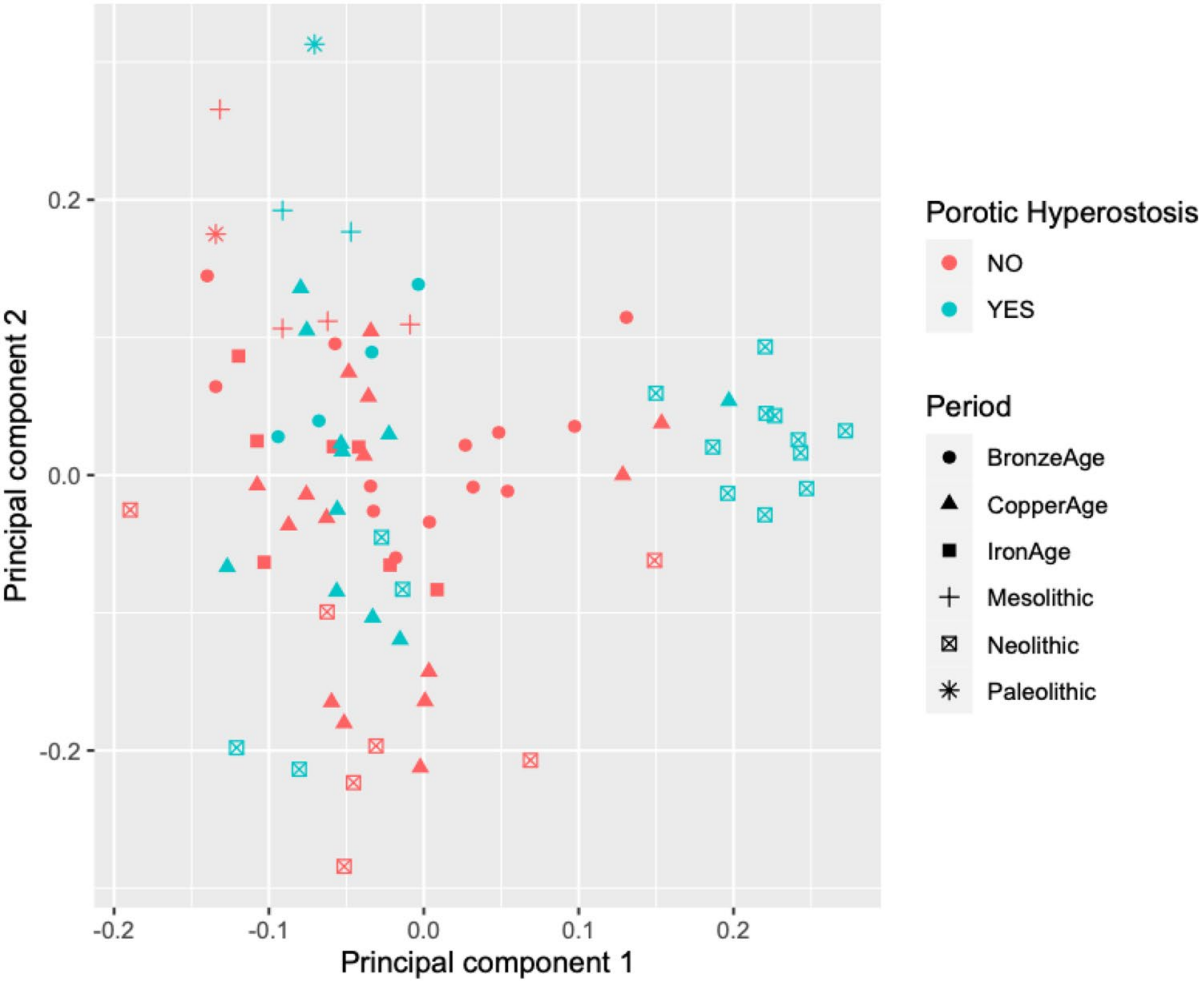
PCA for the 80 ancient individuals tested here using 772,964 SNPs. Each dot represents an individual. In red are marked individuals that were not affected with PH. In green are shown individuals that had PH. The different shapes represent different periods, encompassing from the Palaeolithic to the Bronze Age. The PCA was calculated with PLINK.

There is the possibility that PH and unaffected individuals belong to two different populations, thus sharing different alleles (including those related to BMD and haemoglobin levels). To discard this possibility I conducted a Principal Component Analysis (see Fig. 4; see “Material and methods”). The results show that both groups do not cluster differentially, suggesting that the differences in the genetic risk score are not caused by differences in their ancestry.

### Neolithic individuals had the lowest values for Bone mineral density and low haemoglobin levels

To understand what could have caused such prevalence of PH in the Neolithic, I calculate the mean of the genetic risk scores by time periods (from the Palaeolithic to the Iron Age) (Table 2). The mean genetic risk score of the different phenotypes were calculated for seven Iron Age, 26 Copper Age, 17 Bronze Age, 22 Neolithic, six Mesolithics and two Palaeolithic individuals ignoring if they were or not affected by PH. The genetic risk score for the vitamin B12 metabolism had the lowest mean value in the Palaeolithic. The genetic risk score for skin pigmentation had the lowest mean value in the Bronze Age. The genetic risk score for iron metabolism had the lowest mean value in the Iron Age. However, both genetic risk scores for BMD and for haemoglobin levels show the lowest mean values during the Neolithic. In particular, the genetic risk score for BMD has decreased from the Palaeolithic to the Neolithic, and increased again from the Neolithic to the Iron Age (see Table 2).

**Table 2:** Genetic risk score for the different phenotypes tested here: bone mineral density, skin pigmentation, iron metabolism, haemoglobin levels and vitamin B12 metabolism.

Period	BMD GS***	SKIN GS	IRON GS	H. LEVELS GS***	VIT.B12 GS
Paleolithic	50.96567	59.39306	35.71429	60	**37.5**
Mesolithic	50.57225	59.20039	45.2381	51.66667	45.83333
***Neolithic***	48.83925***	58.21072	47.07792	31.81818***	49.43182
Bronze Age	49.3436	**56.47739**	51.68067	42.35294	43.38235
Copper Age	49.97524	56.48066	45.05495	39.23077	40.38462
Iron Age	51.22624	56.72998	**34.69388**	38.57143	50

In bold script, the lowest value in each phenotype. *** marks the phenotypes where the lowest values coincide with the Neolithic (bone mineral density and haemoglobin levels).

**Figure 5:**
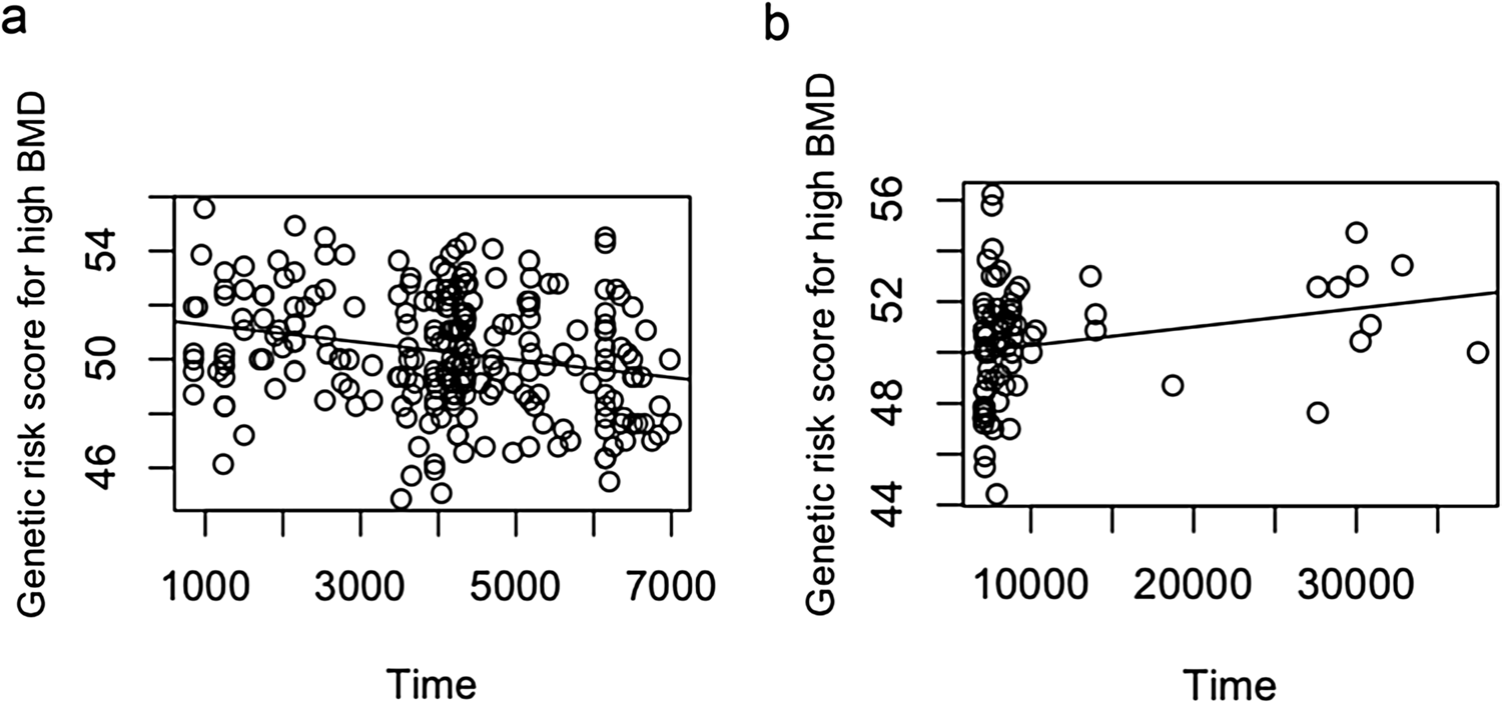
(**a**) A statistical increment in the BMD risk score has been observed since the Early Neolithic to the Iron Age (p value = 0.0002707, R2 = 0.05592). Only individuals younger than 7033.357 years ago are plotted. (**b**) A statistical decay of the BMD risk score has been observed since the Palaeolithic to the Neolithic (p value = 0.04006, R2 = 0.05725). Only individuals older than 7033.357 years ago are plotted. Each dot represents an individual.

This last result is confirmed by studying the genetic risk scores of 308 ancient samples (including the 80 used in the previous analysis) (see “Material and methods”) through time (Fig. 5). The mean value for the age of the Neolithic individuals with PH is 7033.357 years. In order to confirm that BMD decreased from the Palaeolithic to the Neolithic, I compared the genetic risk score for the BMD levels of all the individuals older than 7033.357 years ago.There is a statistical decrease in the genetic risk score for the BMD, reaching the lowest values in the Neolithic period (p value = 0.04006, R2 = 0.05725) (Fig. 5). Additionally, I tested for an increment in the genetic risk score of the BMD, after the Neolithic by comparing all the individuals younger than 7033.357 years ago. The results show a statically increase of the genetic risk score for BMD, with the lowest values being observed in the Neolithic (p value = 0.0002707, R2 = 0.05592). These results suggest that Neolithic individuals had the lowest risk scores for BMD, and can explain why PH had this high prevalence in this specific period compared with previous and later times^2,9–11^.

**Figure 6:**
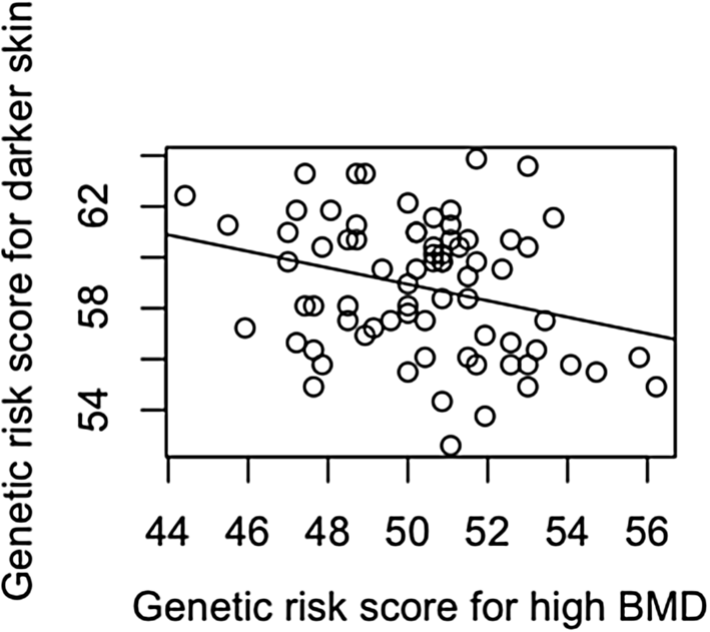
The genetic score for darker skin pigmentation correlates with the genetic score for low BMD levels in 74 ancient individuals belonging to the Early Neolithic and previous periods. In the horizontal axis is the genetic risk score for the BMD, and in the vertical axis is the genetic risk score for darker skin pigmentation. Each dot represents an individual.

The observed decrement in the BMD during the Palaeolithic and Mesolithic is compatible with previous studies, suggesting a selection for a low BMD in Eurasians during the last thousands of years^18,19^. As selection for light skin increased drastically after the Neolithic in Europeans, low BMD levels may have been an adaptation to low UV-radiation in the European latitudes. In this line, I observed a statistical correlation among the genetic risk scores for skin pigmentation and BMD in ancient Mesolithic and Palaeolithic individuals (Fig. 6) (p value = 0.01368, R2 = 0.0815), where low genetic score for BMD correlates with high genetic scores for a darker skin pigmentation. However, this correlation is not observed after the Neolithic (p value = 0.6766, R2 = 0.0007544). Ancient Europeans dating from the Palaeolithic and Mesolithic seem to have had darker skin pigmentation than Neolithic or present-day Europeans^31,32^, thus producing lower levels of vitamin D. Some studies suggest that BMD correlates with low vitamin D levels^50–52^, so it is possible that this reduction in the BMD levels during the Palaeolithic and Mesolithic may be an adaptation to the UV-radiation of the high latitudes of Europe. If this is true, it may explain how ancient Europeans with high melanin production maintained a healthy skeletal system in European latitudes, giving a plausible explanation of the evolutionary reasons of the decrease of BMD in Europeans compared to other populations^18,19^.

## Conclusion

Porotic hyperostosis had a higher prevalence in ancient times than nowadays, specifically during the Neolithic^2,9–11^. It is commonly suggested that anaemia is the main cause for this disease, however, genetic analysis has not been performed in ancient remains. Here I tested several proposed hypotheses for the cause of PH using 80 ancient individuals for which it is known if they had the disease or not, from different periods spanning the Palaeolithic to the Iron Age. While environmental factors can influence the development of PH, the results suggest that the individuals may have already been genetically predisposed for the disease. I do not observe statistical changes in iron or vitamin B12 metabolism, neither in malaria resistant alleles nor in the vitamin D levels. On the other hand, this research suggests that individuals with PH had a genetic disposition to low haemoglobin levels as well as for a low bone mineral density. Both phenotypes are known to correlate, which is likely due to the fact that red blood cells are generated in the diplöe, which grows inside the bone matrix. Interestingly, the low BMD observed in the Neolithic compared to all other periods may explain the highest prevalence of PH during the Neolithic. BMD seems to have decreased from the Palaeolithic to the Neolithic and increased again after the Neolithic. A possible explanation for the decrease is that ancient Europeans adapted to the low UV-radiation by reducing the need in calcium for the bones (reducing the BMD). Selection for light skin was stronger after the Neolithic, so Palaeolothic and Mesolithic individuals had darker skin pigmentation. A still answered question is how these ancient Europeans maintained a healthy skeletal system in the high latitudes of Europe with low UV-radiation if they had high melanin production. Here I suggest that the observed decrease in the BMD genetic score through Palaeolithic and Mesolithic times may be an adaptation to this low vitamin D production.

## Supplementary Information

Supplementary Tables.

## Data Availability

For the main analysis I used data from 47 individuals without PH and 33 individuals who had PH (see Supplementary Table 7). These 80 individuals are part of a dataset of 167 ancient individuals^42^ (see Supplementary Table 8). The genotypes of these individuals are accessible through https://doi.org/10.5061/dryad.b5mkkwhfp. The whole dataset was later used together with another dataset of 153 ancient individuals to track changes in the BMD and Haemoglobin levels in Europeans during the last 40,000 years^43^ (see Supplementary Table 9). The genotypes from the second dataset are accessible through https://zenodo.org/record/7576714#.Y9Qp4OzMKfV. For these analyses I used the data imputed with beagle4.
